# Multidrug-Resistant *Acinetobacter baumannii* Clone, France

**DOI:** 10.3201/eid1905.121618

**Published:** 2013-05

**Authors:** Rémy A. Bonnin, Gaëlle Cuzon, Laurent Poirel, Patrice Nordmann

**Affiliations:** Hôpital de Bicêtre, Kremlin-Bicêtre, France

**Keywords:** Acinetobacter baumannii, carbapenemase, New Delhi metallo-β-lactamase 1, NDM-1, ST85, bacteria, multidrug resistance, France, antimicrobial resistance

**To the Editor:** *Acinetobacter baumannii* is an opportunistic pathogen that is a source of nosocomial infections, mostly pneumonia (*1*). Treatment of infections caused by *A. baumannii* is becoming a serious clinical concern as this microorganism becomes increasingly resistant to multiple antimicrobial drugs (*2*). *A. baumannii* resistance to carbapenems is mostly associated with production of carbapenem-hydrolyzing class D β-lactamases and metallo-β-lactamases (*2*). New Delhi metallo-β-lactamase 1 (NDM-1) is one of the most recently discovered metallo-β-lactamases among various gram-negative species, including *A. baumannii* (*3*). We recently reported the recovery of NDM-1–producing *A. baumannii* isolates throughout Europe (*4*). In that study, the genetic background of several strains was identified and corresponded to sequence types (STs) 1, 25 and 85. The ST85 clone was isolated in France from 2 patients previously hospitalized in Algeria (*4*,*5*).

The present study was initiated by the recent isolation of 6 more NDM-1–producing *A. baumannii* linked with North Africa. To determine the extent of spread of this organism from Africa to France, we genetically analyzed 8 other NDM-1-producing *A. baumannii* isolates collected from different towns in France during 2011–2012. Of these 8 isolates, 6 were from patients previously hospitalized in different cities in Algeria (including Algiers, Setif, Constantine, and Tlemcen), 1 from a patient previously hospitalized in Tunisia, and 1 from a patient previously hospitalized in Egypt. These 8 isolates came from 2 clinical samples (blood cultures and wound) from 6 screening rectal swab samples collected at the time of hospital admission (Technical Appendix). Because the 8 samples were recovered from 5 hospitals, nosocomial acquisition can be ruled out.

The isolates were identified by 16S rRNA gene sequencing. Susceptibility testing was performed by disk diffusion (Sanofi-Diagnostic Pasteur, Marnes-La-Coquette, France) and interpreted according to updated Clinical and Laboratory Standards Institute guidelines (*6*). The MICs of β-lactams (imipenem, meropenem and doripenem) were determined by the Etest technique (AB bioMérieux, Solna, Sweden) according to the manufacturer’s recommendations. All isolates were resistant to β-lactams, including all carbapenems (MICs >32mg/L). The isolates were also resistant to fluoroquinolones, gentamicin, sulfonamides, and chloramphenicol but susceptible to amikacin, netilmicin, rifampin, tetracycline, and tigecycline according to Clinical and Laboratory Standards Institute guidelines (*6*) and colistin according to European Committee on Antimicrobial Susceptibility Testing guidelines (www.eucast.org/fileadmin/src/media/PDFs/EUCAST_files/Disk_test_documents/EUCAST_breakpoints_v1.3_pdf.pdf).

The production of metallo-β-lactamases was suspected by use of a combined disk test, based on the inhibition of the metallo-β-lactamase activity by EDTA as described (*4*). All isolates were positive for production of metallo-β-lactamases.

For all 8 isolates, PCRs aimed at detecting carbapenemase genes, using primers described elsewhere (*7*), followed by sequencing, led to identification of the *bla*_NDM-1_ gene. The isolates also carried a naturally-occurring *bla*_OXA-51_-like gene, namely *bla*_OXA-94_ (Technical Appendix). The *bla*_OXA-51-like_ β-lactamase confers a low level of resistance to carbapenems.

Genotypic comparison was performed by multilocus sequence typing as described (*8*) and by repetitive extragenic palindromic sequence-based PCR by using the DiversiLab system (bioMérieux, La Balme-les-Grottes, France) according to the manufacturer’s instructions. The genomic pattern of all isolates was identical (Figure). Further multilocus sequence typing indicated that all isolates belonged to ST85. This ST was identified in Greece during a nationwide study that focused on carbapenem resistance in clinical isolates of *A. baumannii* and identified mainly carbapenem-hydrolyzing carbapenemase OXA-58 (*9*).

**Figure:**
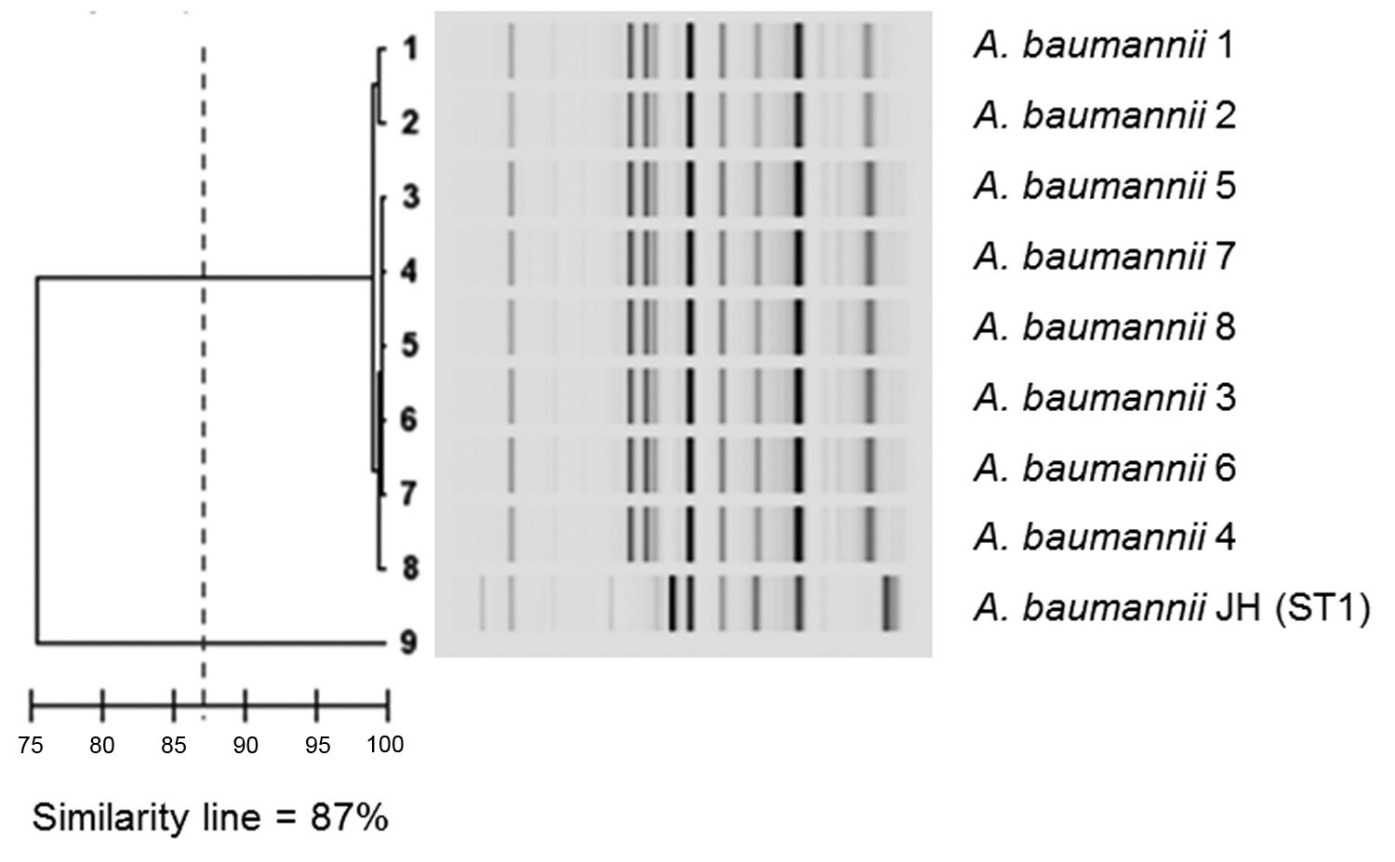
Results of Diversilab system (bioMérieux, La Balme-les-Grottes, France) analysis of *Acinetobacter baumannii* isolates. Similarity line shows the cutoff that separates the different clones.

Recently, we showed that the *bla*_NDM-1_ gene was carried by a composite transposon bracketed by 2 copies of IS*Aba125* in *A. baumannii* (*10*). Cloning and sequencing of the genetic context of the *bla*_NDM-1_ in the first isolate showed that transposon Tn*125* was truncated at its 3′-end extremity by insertion sequence IS*Aba14*, giving rise to a truncated Tn*125* (ΔTn*125*). PCR mapping of all isolates showed that they possessed this truncated isoform of Tn*125*, which was therefore probably no longer functional. 

The identification of several clinical *A. baumannii* isolates that possessed the *bla*_NDM-1_ gene and originated from North Africa, with no obvious link to the Indian subcontinent, strongly suggests that 1 NDM-producing *A. baumannii* clone is probably widespread in North Africa and that it might now act as a reservoir for NDM-1. This finding might indicate that control of spread of multidrug-resistant *A. baumannii* would have a primary role in controlling spread of NDM-1.

Technical Appendix Clinical features of New Delhi metallo-β-lactamase–producing *Acinetobacter baumannii.*
