# Supplementary material for: Multidrug-Resistant Acinetobacter baumannii Clone, France
Source: Emerg Infect Dis. 2013 May;19(5):822–3. doi: 10.3201/eid1905.121618 (PMC3647512; doi:10.3201/eid1905.121618)
Supplement: Technical Appendix — Clinical features of New Delhi metallo-β-lactamase–producing Acinetobacter baumannii. [file 12-1618-Techapp-s1.pdf]

Article DOI: http://dx.doi.org/10.3201/eid1905.121618

Multidrug-Resistant *Acinetobacter baumannii* Clone, France

Technical Appendix

Technical Appendix Table. Clinical features of New Delhi metallo-β-lactamase–producing *Acinetobacter baumannii*

Isolate	Origin of patient	Date of isolation	Specimen
1 (Ora-1)	Oran, Algeria	2011 Jul	Rectal swab and blood culture
2 (StN)	Constantine, Algeria	2011 Aug	Rectal swab
3 (112)	Tlemcen, Algeria	2011 Dec	Wound
4 (HAM)	Unknown, Algeria	2012 Apr	Rectal swab
5 (AYA)	Setif, Algeria	2012 Sep	Rectal swab
6 (BOU)	Unknown, Tunisia	2012 Aug	Rectal swab
7 (BER)	Alger, Algeria	2012 Sep	Rectal swab
8 (ABO)	Gizeh, Egypt	2012 Sep	Blood culture
